# Investigation of Luminescence Characteristics of Osmium(II) Complexes in the Presence of Heparin Polyanions

**DOI:** 10.1155/2013/419716

**Published:** 2013-07-16

**Authors:** Yixi Xie, Yu Lei, Shalini Shah, Hao Wu, Jian Wu, Elise Megehee, Enju Wang

**Affiliations:** Department of Chemistry, St. John's University, Queens, NY 11439, USA

## Abstract

The luminescence characteristics of six osmium carbonyl complexes with phenanthroline (phen) or bipyridine (bpy) and pyridine (py), 4-phenylpyridine (4-phpy), or triphenylphosphine (PPh_3_) complexes in the presence of polyanion heparin were studied in both ethanol and aqueous solutions. The influence of heparin on the luminescence of the complexes is heavily dependent on the type of ligands in the complexes and the solvent used. In the ethanol solutions, the heparin solution enhanced the luminescence of the five osmium complexes, with the strongest enhancement to the 4-phenylpyridine complexes; linear curves were obtained in the luminescence enhancement ratio (*F*/*F*_0_) versus the heparin concentration range of 1–40 **μ**g/mL. In aqueous solutions, heparin quenching of the complexes was more significant; a linear quenching curve was obtained with [Os(phen)_2_CO(PPh_3_)](PF_6_)_2_ in the lower concentration range of 1–12 **μ**g/mL. The interaction of these complexes with heparin in the solutions is discussed. The complexes are shown to be successful in the fast and sensitive detection of heparin in commercial injectable samples.

## 1. Introduction

Heparina, a highly sulfated glycosaminoglycan, has the highest negative charge density of any known natural biological molecule [1]. Heparin has been extensively used as an injectable anticoagulant in many clinical procedures for the prevention of blood clotting, especially during open heart surgery [2, 3]. Pharmaceutical-grade heparin is derived from mucosal tissues of slaughtered meat animals such as porcine intestine and bovine lung [4]. Native heparin is a polymer with a molecular weight ranging from 3,000 to 50,000, and the average molecular weight of most commercially prepared heparin is in the range of 12,000 to 15,000. Under physiological conditions, the sulfate groups are deprotonated and attract positively charged counterions (usually sodium) to form a heparin salt. It is in this form that heparin is usually administered as an anticoagulant [5, 6]. At the end of the medical procedure, the heparin has to be rendered inactive to prevent possible bleeding problems. Therefore, clinicians need to know the exact concentration of heparin remaining when they attempt to neutralize the anticoagulant activity of heparin via precise doses of protamine. The current heparin detection method used in hospital is based on the blood clotting time [7, 8] and varies widely due to variation in the blood content of the different individuals. Thus, the discovery of accurate, rapid, reliable safe, and low-cost methods of heparin detection remains an important area of sensor research. For example, electrochemical sensors [9, 10] and optical sensors [11, 12] were intensively studies in the 1990s, and more recent studies have focused on fluorescent spectroscopy [13, 14] and UV/Vis spectroscopy using gold nanoparticles [15]. 

Luminescence spectroscopy is an analytical method that studies the luminescence emission from a chromophore after their electrons are excited from the ground state to a higher energy level. One of the most attractive features of luminescence spectroscopy as an analytical method is its inherent high sensitivity, wide linear response range, and fast detection speed [16]. However, the luminescence intensity of the chromophore (or excited state complex) can be seriously reduced by collision with other chemical species or enhanced by chemical interactions. Through studying the effect of these chemical species on the luminescence intensity of the chromophore, the quantitative determinations of a variety of important inorganic and organic species in trace amounts have been realized by photoluminescence methods [16]. 

Recently, a series of Os(II) carbonyl (CO) complexes with two bipyridine or phenanthroline ligands and one pyridyl or triphenylphosphine ligand as the five other ligands have been synthesized in our lab. The osmium centers in these complexes are all in the +2 oxidation state with an overall +2 charge of the complex ion, and exhibit high emission intensity in the visible region [17, 18]. This research focuses on studying the effect of the heparin polyanion on the photoluminescence of these polypyridyl osmium(II) complexes, for the purpose of finding potential luminescence markers for the detection of the optically nonactive polyanion heparin. To our surprise, polyanions show unique influences on the luminescence spectra and intensity of these osmium complexes. The 1,10-phenanthroline (phen) complexes were compared with the 2,2′-bipyridine (bpy) complexes. For each of these chromophoric ligands, three analogous sets were compared using pyridine (py), triphenylphosphine (PPh_3_), or 4-phenylpyridine (4-phpy) as the sixth ligand. These ligands showed a significant impact on the luminescence characteristics of the osmium complex in the absence as well as in the presence of polyanions. The results of the studies could lead to a new, potential effective detection method for heparin monitoring. 

## 2. Experimental

### 2.1. Material and Reagents

**Figure 1:**
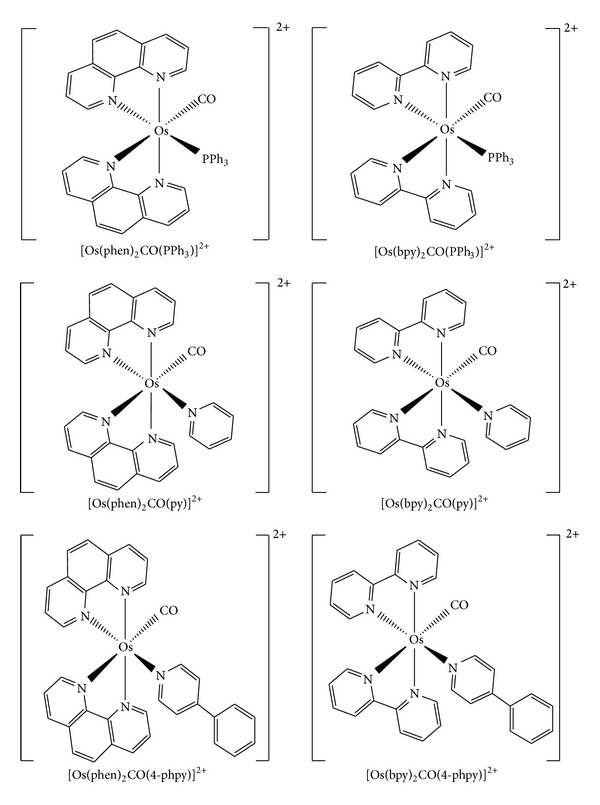
Structures of the six osmium complexes.

The six new Os(II) complexes are [Os(phen)_2_CO(PPh_3_)](PF_6_)_2_, [Os(phen)_2_CO(py)](PF_6_)_2_, [Os(phen)_2_CO(4-phpy)](PF_6_)_2_, [Os(bpy)_2_CO(PPh_3_)](PF_6_)_2_, [Os(bpy)_2_CO(py)](PF_6_)_2_, and [Os(bpy)_2_CO(4-phpy)](PF_6_)_2_. The structure of each complex is shown in Figure 1. The synthesis of six osmium complexes was described in [17, 18]. The purity is >98%.

Heparin sodium sulfate (>180 USP units/mg), ethanol (99.5+%), reagent or analytical grade NaClO_4_, MgSO_4_, CH_3_COONa, NaCl, KBr, NaNO_3_, NaOH, NaNO_2_, KSCN, and KI were all obtained from Sigma-Aldrich. Injectable heparin solutions, 100 units/mL and 40 units/mL with 5% dextrose, were purchased from B. Braun Medical Inc. (Irving, CA, USA). 

### 2.2. Apparatus

Luminescence measurements were made on a Perkin Elmer luminescence spectrometer LS 50 B equipped with a high-intensity xenon flash lamp. A Branson 1210 sonicator was used to help dissolve the osmium complex in solvent when needed. Thermo Scientific digital pipets were used for the addition of all solutions into the quartz cuvette.

### 2.3. Stock Solutions

The stock solutions of the osmium complexes were prepared by dissolving 0.6–2.8 mg of each osmium complex in ethanol (ethanol solution) or distilled water (aqueous solution) in a 100 mL volumetric flask. The 1.0 mg/mL heparin aqueous solution was prepared by dissolving 5.0 mg of heparin in 5.00 mL of distilled water. The 0.10 mg/mL and 0.010 mg/mL heparin aqueous solutions were diluted from the 1.0 mg/mL stock solution. For small anions solutions, a 0.100 M solution of each salts was first made and then diluted to 0.0100 M and 0.00100 M.

### 2.4. Luminescence Measurements

For the luminescence measurements of each osmium complex solution (ethanol, aqueous), 2 mL stock solution was transferred to a quartz cuvette (1 cm × 1 cm × 4.4 cm) placed in the luminescent spectrometer cuvette holder. The emission and excitation slits were both set at 10 nm for the luminescence measurements. A prescan was taken first to obtain the wavelengths of the maximum excitation and emission intensities; then the emission spectrum was recorded by exciting the sample at the appropriate excitation wavelength. In order to study the anion quenching or enhancement of the luminescence of each osmium complex solution, 2.00 mL of the stock solution was placed in the cuvette, and the emission spectrum was measured; then a small amount (10 *μ*L or 20 *μ*L) of the anion solution (0.010~1.0 mg/mL for heparin and 0.10 to 0.0010 M for small anions) was added to the cuvette and the emission spectrum was recorded after the solution was well mixed. The anion solution was continuously added to the cuvette, and the emission spectrum was recorded after each addition. The values of the luminescence intensity were taken at a fixed wavelength at or near the emission maximum. All the luminescence measurements were taken under ambient conditions (~23°C).

Detection of injectable heparin was performed using standard addition method. First, 10 or 20 *μ*L of injectable heparin solution was added to 2 mL of the aqueous [Os(phen)_2_CO(4-phpy)](PF_6_)_2_ solution. Next three portions of 5 *μ*L of 1 mg/mL standard heparin solution were added, and the emission spectrum of the solution was measured before and after each addition. The concentrations of the heparin in the injectable solution were calculated using the standard addition curve.

## 3. Results and Discussion

### 3.1. Excitation and Emission Spectra of Osmium Complexes

**Figure 2:**
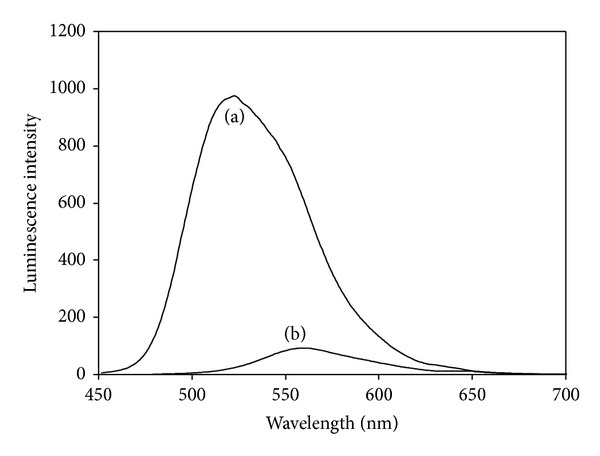
Luminescence spectra of (a) [Os(phen)_2_CO(PPh_3_)](PF_6_)_2_ and (b) [Os(phen)_2_CO(py)](PF_6_)_2_ in ethanol solution (2.4 mg/100 mL).

The excitation and emission spectra of each complex in their ethanol solution (except for [Os(bpy)_2_CO(py)](PF_6_)_2_ which could not dissolve in ethanol) and their aqueous solution were first recorded at room temperature under ambient air. All the spectra exhibited broad emission and excitation bands up to 200 nm in width.  Figure 2 shows the emission spectra in ethanol solution of the two complexes: [Os(phen)_2_CO(PPh_3_)](PF_6_)_2_, and [Os(phen)_2_CO(py)](PF_6_)_2_, which exibit the largest difference in luminescence intensity.

As shown in Table 1 and Figure 2, the two Os-PPh_3_ complexes have much higher luminescence intensities and shorter emission wavelengths than the two Os-(4-phpy) complexes and the two Os-py complexes (520 nm versus 560 nm). The shorter wavelength (higher energy) of the emission is caused by the *π*-backbonding ability of the phosphine which stabilizes the HOMO of the molecule relative to pyridine. The higher luminescence intensities are attributed to the larger steric effect of the PPh_3_ ligand which shields the complex from colliding with the solvent. This slows down the vibrational relaxation to the ground vibrational level of the excited state that must occur prior to emission of a photon of light that occurs when the electron returns to the ground electronic state. All these six complexes exhibit higher luminescence intensities in aqueous solution than in ethanol solution due to the hydrophobicity of the ligands surrounding the osmium center. 

The spectral changes that occur before and after the addition of small inorganic anion solutions suggest that those anions ClO_4_^−^, SO_4_^2−^, CH_3_COO^−^, Cl^−^, Br^−^ and NO_3_^−^ exhibit very little quenching of the luminescence of the osmium complex with anion concentrations up to 0.01 M, while NO_2_^−^, SCN^−^ and I^−^ anions can dramatically quench the luminescence of the osmium complexes with concentrations at 0.01 mM. However, OH^−^ exhibits a small enhancement of the luminescence of the osmium complex; luminescence intensity slightly increased as the solution pH increased from 6 to 9 in aqueous solution. 

### 3.2. Influence of the Luminescence Spectra of Osmium Complexes by Heparin Polyanions

It was expected that heparin would quench the luminescence of the osmium complexes due to its high negative charges. Surprisingly, however, it was found that heparin actually enhanced the luminescence intensity of the osmium complexes in ethanol solutions. The luminescence of each complex in the ethanol and aqueous solution changed quite differently after heparin was added. 

#### 3.2.1. Ethanol Solution

In ethanol solution, each complex exhibited an increase in luminescence intensity after heparin was added in concentrations greater than 0.5 *μ*g/mL. However, the amount of increase varied significantly for each complex. The solutions of [Os(bpy)_2_CO(4-phpy)](PF_6_)_2_, [Os(phen)_2_CO(4-phpy)](PF_6_)_2_, and [Os(phen)_2_CO(py)](PF_6_)_2_ exhibited a very large increase in luminescence intensity while the solutions of [Os(phen)_2_CO(PPh_3_)](PF_6_)_2_ and [Os(bpy)_2_CO(PPh_3_)](PF_6_)_2_ exhibited a small luminescence increase. 

**Figure 3:**
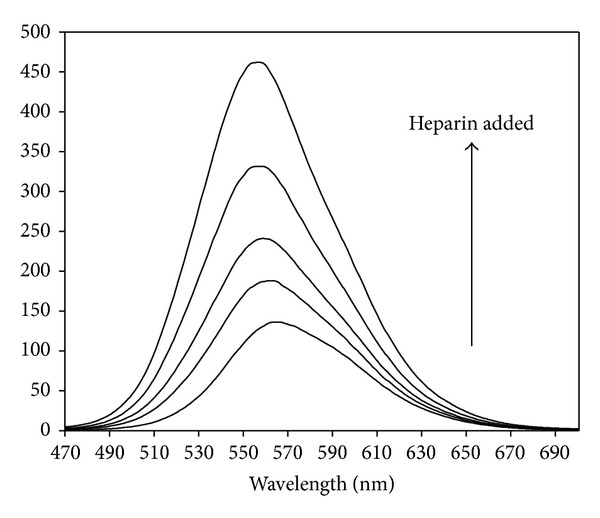
Luminescence spectra of [Os(bpy)_2_CO(4-phpy)](PF_6_)_2_ in ethanol solution (2.8 mg/100 mL) in the presence of varing concentrations of heparin. From bottom to top, heparin concentration: 0, 9.9, 19.6, 29.1, and 38.5 *μ*g/mL.

**Figure 4:**
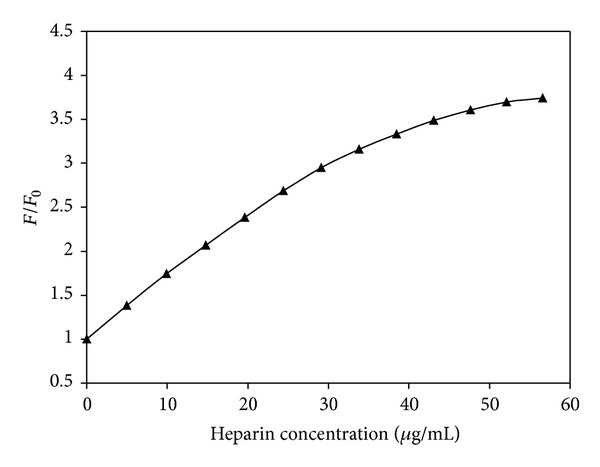
Plot of the luminescence enhancement ratio (*F*/*F*_0_) of [Os(bpy)_2_CO(4-phpy)](PF_6_)_2_ at 560 nm in ethanol solution (2.8 mg/100 mL) versus heparin concentration.

Figure 3 shows the luminescence spectra of the ethanol solution of [Os(bpy)_2_CO(4-phpy)](PF_6_)_2_ in the absence and presence of heparin at different concentrations. Addition of heparin to the solution significantly enhanced the luminescence. The luminescence intensity was increased from a relative emission intensity of 136 in the absence of heparin to 462 by addition of heparin in a concentration range of 5.0–38.5 *μ*g/mL. Further, there is an 8 nm blue shift in wavelength of the emission maxima with high heparin concentrations. A plot showing the wider range of heparin concentrations on the enhancement ratio (*F*/*F*_0_) is shown in Figure 4. Here, *F* and *F*_0_ are the luminescence (fluorescence) intensity of the osmium complexes solutions at 560 nm with and without heparin, respectively. The luminescence intensity increased steadily up to a heparin concentration of 56 *μ*g/mL. In the lower concentration range (up to 30 *μ*g/mL), there is a linear relationship between the *F*/*F*_0_ ratio and the concentration with an *R* ^2^ of 0.99. The heparin enhancement of the [Os(phen)_2_CO(4-phpy)](PF_6_)_2_ (2.4 mg/100 mL EtOH) luminescence has a similar pattern to that seen with [Os(bpy)_2_CO(4-phpy)](PF_6_)_2_. In this case, the luminescence was enhanced by three and half times when the added heparin concentration was increased to 50 *μ*g/mL. Once again, there is an 8 nm blue shift of the emission maxima at higher heparin concentrations. In the concentration range of 1 to 40 *μ*g/mL, there is a linear relationship between the *F*/*F*_0_ ratio and the heparin concentration with an *R* ^2^ of 0.99. 

The heparin enhancement of the luminescence of [Os(phen)_2_CO(py)](PF_6_)_2_ (2.4 mg/100 mL EtOH) is smaller than that seen for the 4-phpy complexes. The luminescence intensity was enhanced by two and half times when the added heparin concentration was increased to 20–30 *μ*g/mL, and then the luminescence started to decrease at heparin concentrations in excess of 30 *μ*m/mL. In this case, there is also an 8 nm blue shift of the emission maxima at higher heparin concentrations. 

The heparin enhancement of the luminescence of the [Os(phen)_2_CO(PPh_3_)](PF_6_)_2_ (2.4 mg/100 mL EtOH) and the [Os(bpy)_2_CO(PPh_3_)](PF_6_)_2_ (2.1 mg/100 mL EtOH) solutions is very small compared to the Os-(4-phpy) and Os-py complexes. Here, the shift in the emission maxima is insignificant (less than 2 nm). 

**Figure 5:**
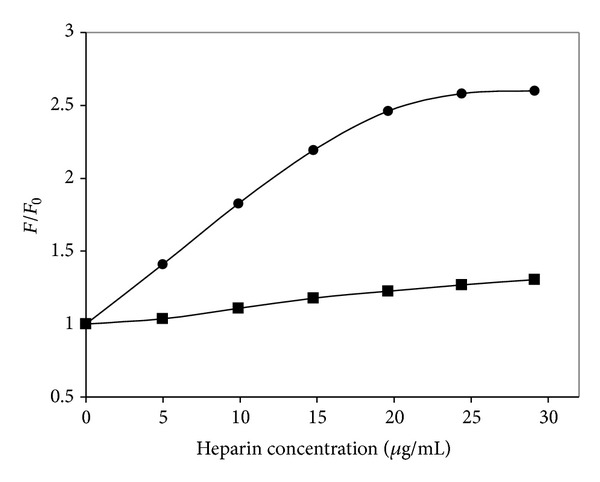
Plot of the luminescence enhancement ratio (*F*/*F*_0_) of (●) [Os(phen)_2_CO(py)](PF_6_)_2_ and (■) [Os(phen)_2_CO(PPh_3_)](PF_6_)_2_ (Ex slit width: 5 nm) both at 2.4 mg/100 mL versus heparin concentration.

Figure 5 shows the plot of *F*/*F*_0_ versus heparin concentration for the two phenanthroline complexes with pyridine and PPh_3_ as the sixth ligand. From Figures 4 and 5, it can be seen that the 4-phpy complexes, which have the lowest solution luminescence in the absence of polyanions, showed the highest enhancement in the concentration range of 5–40 *μ*g/mL added heparin concentrations. By contrast, the PPh_3_ complexes showed the smallest enhancement in the same concentration range. 

**Figure 6:**
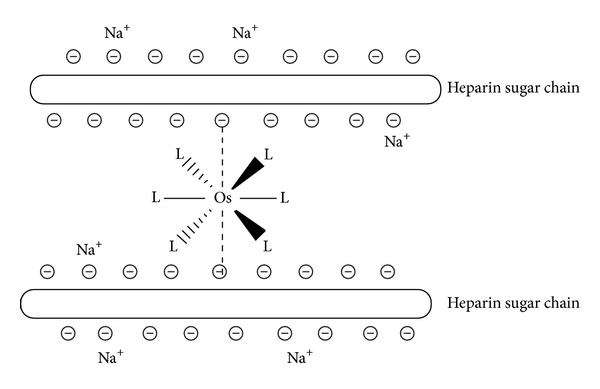
Schematic drawing of the interaction of heparin with the double positive-charged osmium complexes.

The osmium complexes luminescence enhancement by heparin is likely contributed by the close contact of heparin and the complexes, which forms a rigid structure that reduced the solvent collision and quenching. Heparin has a long linear structure with anionic sites on its branches when it is dissolved in aqueous solution [1]. When its aqueous solution is added to the ethanol solution of the osmium complex, the anion sites on its branches attract the double positively charged osmium complex cations. As a result, the osmium complex could electrostatically bind to heparin and become surrounded by the heparin molecules (Figure 6). In this case, solvent molecules can no longer get close enough to the osmium complex to help relax the excited state, resulting in the emission maximum moving to higher energy. This effect would also explain the increased emission intensity since the rate of nonradiative decay would be greatly reduced in the absence of favorable interaction with solvent molecules.

The sixth ligand in these complexes plays an important role in the interaction of these charged sites. The complexes [Os(bpy)_2_CO(4-phpy)]^2+^, [Os(phen)_2_CO(4-phpy)]^2+^, or [Os(phen)_2_CO(py)]^2+^ have small narrow 4-phenylpyridine or pyridine ligand. When heparin is added to their ethanol solution, these osmium complexes could readily approach the heparin polyanion and bind up to two heparin anion sites. These two sites may well form two heparin polyanions branches, thus forming ion pairs between the polyanion and osmium complex. Therefore, heparin can cause significant enhancement to the luminescence of the Os-(4-phpy) and Os-py complexes. Further, the luminescence only stops increasing at very high heparin concentrations when all the osmium complex cations are bound to heparin anion sites. By contrast, the [Os(phen)_2_CO(PPh_3_)]^2+^ and [Os(bpy)_2_CO(PPh_3_)]^2+^ complexes have a sterically bulky PPh_3_ group, which prevents them from approaching the heparin polyanion closely. Therefore, the luminescence intensity of the Os-PPh_3_ complexes could not be significantly enhanced by heparin. 

#### 3.2.2. Aqueous Solution

The effect of heparin on the emission of these osmium complexes in their aqueous solution is quite different from that of their ethanol solution. The aqueous solution of osmium complexes with a PPh_3_ or pyridine group exhibits a decrease in luminescence intensity at low added heparin concentrations; then the luminescence increases at heparin concentration above 15 to 19 *μ*g/mL. However, complexes with the 4-phpy group exhibit a decrease of luminescence at heparin concentrations below 0.5 *μ*g/mL then an increase in luminescence intensity is observed with the addition of heparin in the range of 1 to 15 *μ*g/mL. This shows that as a polyanion, when not binding to the osmium complexes, heparin is an efficient luminescence quencher in aqueous solutions. This is especially true with the PPh_3_ complexes, where binding only occurs at very high (>15 *μ*g/mL) heparin concentrations. By contrast, the 4-phpy complexes bind more strongly to heparin, and so less of a quenching effect is observed.

**Figure 7:**
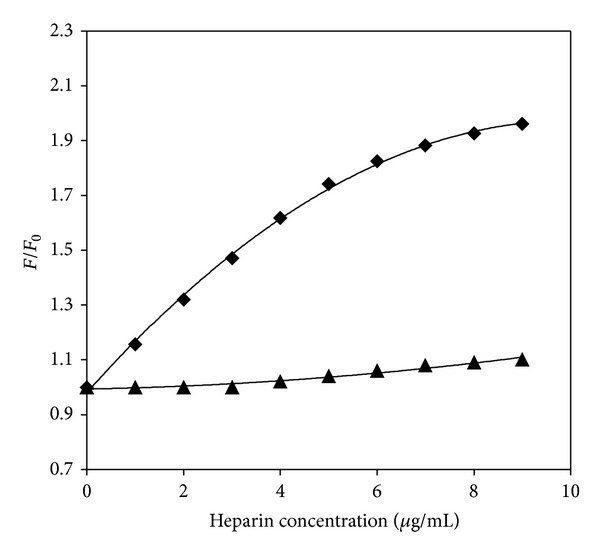
Plots of the luminescence enhancement ratio (*F*/*F*_0_) of (◆) **[**Os(phen)_2_CO(4-phpy)](PF_6_)_2_, and [▴][Os(bpy)_2_CO(4-phpy)](PF_6_)_2_ at 560 nm in aqueous solution (1.2 mg/100 mL) versus heparin concentration.

Figure 7 shows the plots of the luminescence enhancement ratio (*F*/*F*_0_) as a function of heparin concentration for the two Os-(4-phpy) complexes in aqueous solution. The high luminescence enhancement ratio of the 1,10-phenanthroline complexes by heparin shown in Figure 7 suggests that the [Os(phen)_2_CO(4-phpy)]^2+^ complex binds more strongly to the heparin polyanion than the analogous 2,2′-bipyridine complex complexes. Comparing Figures 4, 5, and 7, the enhancement of heparin to the [Os-CO(bpy)2(4-phpy)]^2+^ complex is much smaller in aqueous solutions, which indicate a weaker binding to heparin, possibly due to the higher solubility of heparin and the complexes in aqueous solution. 

In the aqueous solutions of [Os(phen)_2_CO(PPh_3_)](PF_6_)_2_ or [Os(bpy)_2_CO(PPh_3_)](PF_6_)_2_, it is even harder for the osmium complex to bind to heparin because of the high solubility of heparin in aqueous solution and the hindrance of the bulky PPh_3_ group. In fact, at low heparin concentration, heparin quenches the luminescence of these two complexes heavily as polyanion. The decrease of the luminescence intensity in the aqueous solution of these two complexes fits the Stern-Volmer equation

(1) $$\begin{array}{c} \frac{F_{0}}{F}=1+K_{\text{sv}}\left[Q\right]. \end{array}$$

Here *F*_0_ and *F* are the luminescence signals in the absence and in the presence of a quencher, respectively; *K*_sv_ is the Stern-Volmer quenching constant; and [*Q*] is the concentration of the quencher. 

**Figure 8:**
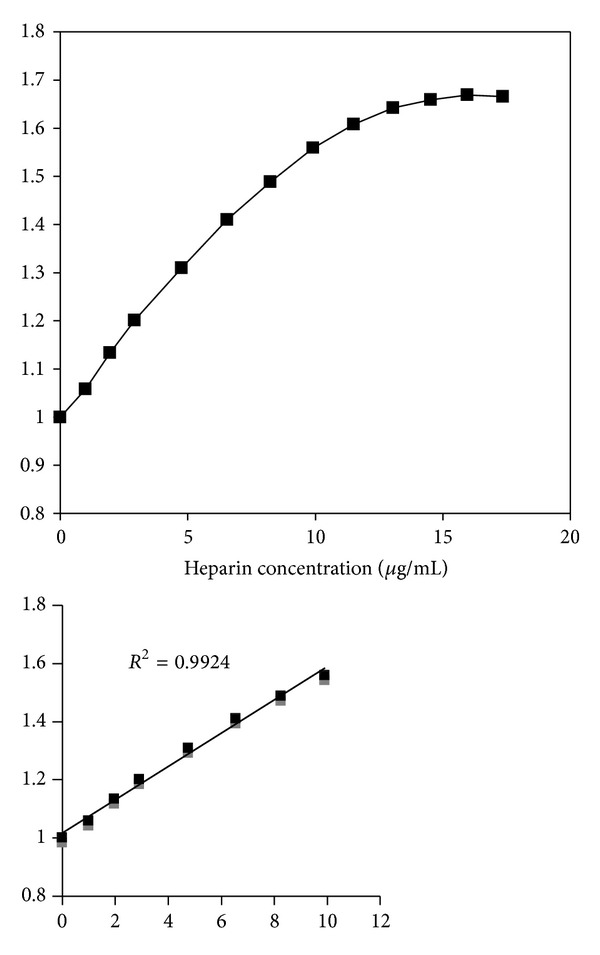
Stern-Volmer plot for the heparin quenching of the luminescence of [Os(phen)_2_CO(PPh_3_)](PF_6_)_2_ aqueous solution (1.2 mg/100 mL in H_2_O).

Stern-Volmer plot is shown in Figure 8 for the [Os(phen)_2_CO(PPh_3_)]^2+^ complex. The high linearity of the Stern-Volmer curve, shown at the concentration range of 1 to 12 *μ*g/mL in the insert, confirms that heparin exhibits dynamic quenching to the Os-PPh_3_ complexes. The nonlinearity at higher concentrations may indicate that osmium complex-heparin ion-pairs have formed. Further addition of heparin above 12 *μ*g/mL caused the luminescence of these complexes to increase slightly.

Table 2 summarizes the heparin concentration ranges that induced significant luminescence changes to the complexes studied in ethanol or aqueous solutions. In ethanol solution, [Os(bpy)_2_CO(4-phpy)](PF_6_)_2_ exhibits the greatest enhancement in the presence of heparin as measured by the highest slope of the *F*/*F*_0_ versus heparin concentration linear curve. This complex exhibits the longest response range and the highest sensitivity to heparin concentration. In aqueous solutions, the [Os(phen)_2_CO(4-phpy)](PF_6_)_2_ and both phosphine complexes showed significant luminescence changes at low heparin concentrations. 

### 3.3. Detection of Heparin in Medicinal Solution

The detection of heparin was then performed with commercial medicinal solutions to evaluate the feasibility. Two commercial heparin sodium injection solutions (40 and 100 units/mL in 5% dextrose, in 100 mL plastic bags) from B. Braun Medical Inc. were tested. With the [Os(phen)_2_CO(4-phpy)]^2+^ complex in aqueous solution, the emission intensities of the osmium solution before and after adding 20 *μ*L of these two heparin solutions were measured. The ratios (*F*/*F*_0_) obtained were 1.30 and 1.97 (*n* = 5), respectively, showing that the osmium complex indeed had a greater response to higher heparin concentration preparations. However, these medical preparation responses are much higher than the pure heparin solution response. When response curve of heparin in the presence of 5% dextrose was performed, an enhanced osmium complex response to heparin concentration was observed. This enhanced response is likely due to the hydrophobic environment that the polysaccharide, dextrose, created around the osmium complex in aqueous solution, thus reducing the extent of nonradiative decay of the excited states and increasing the luminescence intensity of the osmium complex. 

To eliminate the background effect, the standard addition method was used. First, 10 or 20 *μ*L of the commercial heparin sample solutions was added to the aqueous osmium complex solution; then three portions of 5 *μ*L of the 1.0 mg/mL heparin standard solution were added. The luminescence values of the complex solution after each addition of the sample and standard heparin solutions were measured at 560 nm. With the standard addition curves and using the conversion of 180 units/mg of sodium heparin, the average heparin concentration in the two sample bags was calculated to be 46 ± 3 and 111 ± 3 units/mL. The relative standard deviation, RSD for the two corresponding samples are 5.6 (*n* = 3) and 2.3 (*n* = 6) respectively, indicating that the precision of the method is acceptable considering the volume added is very small, although they are 11% higher than the labeled value. Further tests, with other commercial sources having different backgrounds, are currently under study.

## 4. Conclusion

The luminescence of osmium carbonyl complexes is stable at room temperature in ethanol and aqueous solutions, though the intensity and wavelength of this emission are heavily dependent on the other five ligands. The effect of heparin on the luminescence of the osmium complexes depends heavily on both the ligand and solvent used. In ethanol solutions, heparin could enhance the luminescence of five osmium complexes at low heparin concentrations, with the greatest enhancement occurring with the Os-(4-phpy) complexes. This increased emission intensity may be due to binding of heparin to the double charged complexes which changed the solvent environment of the complexes. In aqueous solutions, heparin quenching the osmium complex emission was more significant as heparin is more soluble in water and behaves as an anion quencher. These osmium complexes are shown to be useful for the fast and sensitive detection of heparin in commercially injectable samples.

## Figures and Tables

**Table 1:** Excitation and emission maxima wavelengths (nm) of the six osmium complexes.

Complex	\*lambda*_Em_ in EtOH	\*lambda*_Em_ in H_2_O	\*lambda*_Ex_ in EtOH	\*lambda*_Ex_ in H_2_O
[Os(phen)_2_CO(PPh_3_)](PF_6_)_2_	520	520	396	396
[Os(bpy)_2_CO(PPh_3_)](PF_6_)_2_	520	520	396	396
[Os(phen)_2_CO(4-phpy)](PF_6_)_2_	560	560	420	420
[Os(bpy)_2_CO(4-phpy)](PF_6_)_2_	560	560	430	430
[Os(phen)_2_CO(py)](PF_6_)_2_	560	560	420	420
[Os(bpy)_2_CO(py)](PF_6_)_2_	N/A	560	N/A	420

**Table 2:** Linear response ranges and slopes obtained from the *F*/*F*_0_ curves to heparin of the three osmium complexes.

Complex	Solution	Linear range (*μ*g/mL)	Slope
[Os(bpy)_2_CO(4-phpy)](PF_6_)_2_	Ethanol solution	1–38	0.061
[Os(phen)_2_CO(4-phpy)](PF_6_)_2_	Ethanol solution	1–40	0.072
[Os(phen)_2_CO(py)](PF_6_)_2_	Ethanol solution	1–24	0.074
[Os(phen)_2_CO(4-phpy)](PF_6_)_2_	Aqueous solution	1–10	0.116
[Os(phen)_2_CO(PPh_3_)](PF_6_)_2_	Aqueous solution	1–10	−0.036*

*Value from the Stern-Volmer equation.
